# Metals and Metalloids Accumulation and Biomagnification in Three Commercially Important Fishes from a Turkish Brackish Lake

**DOI:** 10.1007/s00128-026-04187-1

**Published:** 2026-02-13

**Authors:** Paride Balzani, Irmak Kurtul, Esengül Köse, Sadi Aksu, Özgür Emiroğlu, Elif Tuğçe Aksun Tümerkan, Sercan Başkurt, Oğuzcan Mol, Emre Çınar, Ali Serhan Tarkan

**Affiliations:** 1 Faculty of Fisheries and Protection of Waters, South Bohemian Research Centre of Aquaculture and Biodiversity of Hydrocenoses, University of South Bohemia in České Budějovice, Zátiší 728/II, 389 25 Vodňany, Czech Republic; 2 Marine and Inland Waters Sciences and Technology Department, Faculty of Fisheries, Ege University, İzmir, Türkiye; 3 Faculty of Health, Environment and Medical Sciences, Bournemouth University, Poole, Dorset, UK; 4 Department of Environmental Protection Technologies, Eskişehir Vocational School, Eskişehir Osmangazi University, Eskişehir, Türkiye; 5 Vocational School of Health Services, Eskişehir Osmangazi University, Eskişehir, Türkiye; 6 Department of Biology, Faculty of Science, Eskişehir Osmangazi University, Eskişehir, Türkiye; 7 Department of Food Processing-Food Technology, Vocational School of Health Services, Ankara Yıldırım Beyazıt University, Ankara, Türkiye; 8 Department of Ecology and Vertebrate Zoology, Faculty of Biology and Environmental Protection, University of Lodz, Lodz, Poland

**Keywords:** Biomagnification, Bioaccumulation, Pollution, Food web, Fisheries, Lagoon, Aegean sea

## Abstract

Metal and metalloid (hereafter simply ‘metal’) pollution is a growing global concern for both aquatic organisms and human health. Fishes are important in human diet, but they can also accumulate metals in their tissues. Therefore, it is important to monitor the concentration of metals in their muscle, and to investigate potential patterns of bioaccumulation and biomagnification. Here, we studied the bioaccumulation and biomagnification of chromium (Cr), nickel (Ni), arsenic (As), and lead (Pb) in three economically important fish species from Dalyan Lake (Türkiye): the grey mullet *Mugil cephalus*, the gilthead seabream *Sparus aurata*, and the European seabass *Dicentrarchus labrax*. To assess trophic transfer patterns, we combined metal and carbon and nitrogen stable isotope analyses. The trophic niches of the three species were differentiated, with *D. labrax* occupying a higher trophic position compared to the other species. We found significant interspecific differences in metal accumulation, especially due to different Pb, As, and Cr concentrations. The concentration of As was highest in *S. aurata* (2.07 µg/kg), intermediate in *M. cephalus* (1.49 µg/kg), and the lowest in *D. labrax* (1.06 µg/kg), possibly reflecting a greater exposure to polluted sediment in *S. aurata*. The concentration of the other metals were similar among species, ranging from 1.47 (*S. aurata*) to 1.58 (*M. cephalus*) µg/kg for Cr, from 0.51 (*S. aurata* and *M. cephalus*) to 0.56 (*D. labrax*) µg/kg for Ni, and from 0.25 (*D. labrax*) to 0.47 (*M. cephalus*) µg/kg for Pb. Despite no significant differences among species were found in its concentration, Cr showed evidence of biomagnification. The concentration of As, Ni, and Pb did not show biomagnification, but rather trophic dilution. This study demonstrates the value of integrating ecological tracers with contaminant analysis to better understand pollutant dynamics in aquatic food webs.

## Introduction

Aquatic pollution is one of the most pressing environmental hazards nowadays (e.g. Köse et al. 2023; Let et al. 2025). Among aquatic pollutants, metals and metalloids (hereafter ‘metal’ for simplicity) play a major role due to their toxicity, persistence, and bioavailability (Gall et al. 2015; Zhang et al. 2018). While metal concentrations in water can be of natural origins, such as rock weathering and volcanic activity, anthropogenic inputs —including industrial discharges, urban runoff, agriculture, and mining — are increasingly contributing (Munir et al. 2022; Hossen and Mostafa 2023; Angon et al. 2024).

Once uptaken from the environment, some metals can bioaccumulate in aquatic animal tissues over time, causing adverse biological effects (Wang and Tan 2019; Balzani et al. 2022; Jamil Emon et al. 2023). The extent of accumulation depends on several factors, including species, age, body size, and environmental conditions such as pH and temperature (Mason et al. 1996; Yi et al. 2011; Balzani et al. 2022). Besides evironmental factors, the species’ biology and ecology is of primary importance in accumulation patterns. Indeed, since bioaccumulation can occur from both the water and the sediment, it can differ between benthic/littoral and pelagic habitats (Ciesielski et al. 2016). At the same time, metal accumulation could come from contaminated food sources, thus interspecific differences in the trophic ecology, both in the diet and feeding habitat, could lead to different accumulation patterns (Feng et al. 2021). Moreover, some metals can biomagnify, i.e. increase their concentration towards higher trophic levels (Markert et al. 2003; Madgett et al. 2021; Yang et al. 2021). Therefore, metal pollution could represent a serious ecological and public health issue (Penigrahi et al. 2021; Shahjahan et al. 2022).

Fish are especially relevant in this context, not only because they concentrate pollutants, but also because they bridge multiple trophic levels, playing a central role in contaminant transfer (Balzani et al. 2021; Köse et al. 2023). Moreover, they are one of the most important food sources for humans, being highly demanded due to their high nutritional values (Taylor et al. 2019; Ahmed et al. 2022; Oficialdegui et al. 2025), with fisheries being one of the most important economic sectors worldwide (Farquhar et al. 2024; Basurto et al. 2025). Given the high human consumption of fish, it is thus important to assess the metal concentration in the fish species that enter in the market (Samantara et al. 2023; Prabakaram et al. 2024; Zhou 2025).

One very useful approach to investigate the patterns of metal accumulation and biomagnification is the combined application of metal and stable isotope analysis (Jardine et al. 2006; Lavoie et al. 2013). Stable isotope analysis provides a long-term and time-mediated information on consumed resources, owing to predictable changes in isotopic ratios between the prey and its consumer (Boecklen et al. 2011). Thus, it allows to trace feeding relationships and characterize trophic niches using isotopic markers, the most common of which are δ^15^N (proxying the trophic position) and δ^13^C (proxying the main C source and the feeding habitat) (Post 2002; Layman et al. 2012). Thus, integrating these two analytical techniques provides a better understanding of interspecific differences in accumulation patterns, as well as of the biomagnification of metals, by tracking them across trophic positions (Watanabe et al. 2008; Balzani et al. 2021). This integrated approach has been applied globally to both freshwater and marine ecosystems (e.g. Sun et al. 2017; Lau and Le 2023). However, similar applications remain rare in Türkiye (but see e.g. Mutlu 2021), thus this study helps address that gap by applying well-established international methods to local fish communities, offering new insight into contaminant transfer in Turkish waters.

In this study, we applied this integrative approach to a Turkish brackish lake ecosystem to explore how metals accumulate and potentially biomagnify. We assessed the concentration of four metals (Cr, Ni, As, and Pb) in three species of commercially important fish belonging to different trophic guilds (the omnivorous flathead grey mullet *Mugil cephalus* Linnaeus, 1758, the predatory gilthead seabream *Sparus aurata* Linnaeus, 1758, and the top predator European seabass *Dicentrarchus labrax* (Linnaeus, 1758)). Specifically, we tested whether (i) different species show different metal accumulation patterns, (ii) different species occupy distinct trophic niches based on their feeding habitat (proxied by δ^13^C) and trophic position (proxied by δ^15^N), (iii) metal accumulation relates to the feeding habitat or trophic position, and (iv) biomagnification occurs in the studied ecosystem. We hypothesized (i) a different accumulation pattern based on the different species’ ecology, with *M. cephalus* occupying and feeding in more coastal habitats compared to the other species and all species occupying different trophic levels, thus also ii) having segregated trophic niches. We also hypothesized (iii) significant positive relationships between metal concentrations and both δ^13^C and δ^15^N, mostly driven by the more coastal habits of *M. cephalus*, and to the different trophic positions of the studied species. As such, we also hypothesized (iv) biomagnification to occur for all metals in the studied ecosystem.

## Materials and methods

### Study Site and Sampling

Lake Dalyan (40.719344 N, 26.070564 E, Edirne province) is a shallow lagoon on the European side of Türkiye connected to the Aegean Sea with a narrow channel (Figure S1). This lake is located close to the mouth of the Meriç River, a major transboudary river (~ 480 km long) forming the border with Bulgaria and Greece. The lake was formed by the river’s alluvial deposits and supports rich aquatic biodiversity, making it an important habitat for fish and especially for waterfowl species. The hydrology of the lake is determined by marine intrusion, river discharge, and local precipitation, resulting in variable salinity conditions. The region has a Mediterranean climate, with warm-hot summers (24–28 °C) and mild winters with most annual rainfall. The surrounding landscape is dominated by agricultural land (primarily crop fields), small fishing settlements, and limited urban development, providing multiple potential pathways for nutrient, sediment, and contaminant inputs into the lake (Tokatlı and Ustaoglu 2020; Tokatlı and Islam 2022; Aydın 2024; Köse 2025).

The most abundant and commercially important fish species present in the lake (*S. aurata*, *D. labrax*, and *M. cephalus*) were sampled during summer 2021, using multi-mesh gillnets with 12 panels of mesh sizes ranging 5–55 mm, at a depth of around 2 m. The nets were in place for 12 h, from 18:00 to 6:00. Overall, 27 individuals (9 for each species) were caught. Collected specimens were stored in a cool box containing ice (4 °C) and transported to the laboratory. After recording the total length (TL, accuracy 1 mm) and total weight (TW, 0.1 g) of each specimen, two samples of the dorsal muscle of each individual were taken, one for the metal analysis and one for C and N stable isotope analysis. Muscle samples were preserved without any chemical in the freezer at – 20 °C until further processing.

### Metal Analysis

Muscle samples for metal analysis were dehydrated at 105 °C for 3 h and then weighed. Then, samples were digested using microwave-assisted mineralization (Lao et al. 2023; Varol et al. 2022), by placing 0.20–0.25 g (dry weight) of muscle into a digestion vessel containing 8 ml of concentrated nitric acid (HNO_3_) and 1 ml of hydrogen peroxide (H_2_O_2_), and then heated at 200 °C for 30 min in a microwave digestion unit (CEM Mars Xpress) (Tokatlı and Islam 2022). After organic destruction, the samples were allowed to cool to room temperature and adjusted to a final volume of 50 ml with ultrapure water (Köse et al. 2020; Tokatlı and Islam 2022).

The concentration of four metals (Cr, Ni, As, and Pb) in muscle samples was measured by Inductively Coupled Plasma – Mass Spectrometry (ICP-MS) as means of triple reads in the Eskişehir Osmangazi University Center Research Laboratory Application and Research Center. The respective mean metal concentration was used for the statistical analyses. A total of 5 blanks (one every approximately 5 samples) were also prepared to control for contamination. In the ICP-MS device, calibration programs were prepared with solutions in the concentration range of 0.001–1 µg/ml (ppm) from the reference standards (NIST SRM, National Institute of Standards and Technology) of the elements to be analyzed. The relative standard deviation was 1–5% for all metals. The limit of detection (LOD) for the four metals were: Cr = 0.0000621, Ni = 0.0000083, As = 0.0000058, Pb = 0.0000038.

### Stable Isotope Analysis

Muscle samples for C and N stable isotope analysis were dehydrated in oven at 60 °C and then ground to a fine powder using an agate pestle and mortar to homogenize the sample. At the Akdeniz University Food Safety and Agricultural Research Centre in Türkiye, approximately 1 (± 0.1) mg of each homogenized sample was weighed into tin capsules and analyzed using an isotopic ratio mass spectrometer (Delta V Advantage, Thermo Fisher Scientific, Germany) in conjunction with a continuous flow interface (ConFlo IV, Thermo Fisher Scientific, Germany). Results were expressed using the δ notation (‰): δ^13^C or δ^15^N = ((Rsample/Rstandard) − 1) × 1000, where R stands for the ^13^C:^12^ or ^15^ N:^14^N ratios. For C and N, respectively, the standards were air N_2_ and Vienna Pee Dee Belemnite. Samples were analyzed in duplicates to guarantee a good reliability (the average standard errors for δ^13^C and δ^15^N were 0.03 and 0.11, respectively), and the average for each sample was used for the statistical analyses.

### Statistical Analyses

Before statistical analysis, each value of samples that presented metal concentrations below the detection limit was substituted with the value of the detection limit itself (Balzani et al. 2021). Then, to account for the different quantities of sample analyzed, the values were converted to µg/kg using the formula: Mw = (Mv × V) × 1000 / W, where Mw is the metal concentration in µg/kg, Mv is the metal concentration in mg/l, V is the volume of the sample (in ml), and W is the sample dry weight (in g). The obtained metal concentrations (in µg/kg) were log_10_ transformed to account for multiplicative effects.

Differences in the metal concentrations (using the log_10_ transformed data) among species were tested with a permutational multivariate analysis of variance (PERMANOVA) using Bray–Curtis dissimilarity index and 9999 permutations, using the function *adonis2* of the R package vegan (Oksanen et al. 2024). A post hoc test with Benjamini-Hochberg P value correction for multiple comparisons was performed using the wrapper function *pairwise.adonis* (Arbizu 2020). These differences were represented with a non-metric multidimensional scaling (nMDS) using the function *metaMDS* of the R package vegan, on which the significant vectors of the metal concentrations were plotted using the function *envfit* of the same R package (Oksanen et al. 2024). Then, we used the function *dbrda* of the R package vegan to perform a distance-based redundancy analysis (db-RDA) on the Bray-Curtis dissimilarity matrix, with the log_10_-transformed metal concentrations as explanatory variables. A forward variable selection over a null model was used to identify the significant metal concentrations, using the function *ordistep* (9999 permutations) of the R package vegan. The final db-RDA was then tested using an analysis of variance (ANOVA) followed by Benjamini–Hochberg *P* value correction for multiple comparisons. We then tested the differences in each metal concentration (log_10_ transformed) among species using a series of Kruskal–Wallis tests, followed by Mann–Whitney post hoc tests. To assess differences in the isotopic niche, we performed a PERMANOVA (with Euclidean distance and 9999 permutations) on the δ^15^N and δ^13^C, followed by a post hoc test with Benjamini-Hochberg *P* value correction for multiple comparisons. To test for which stable isotope the niches differed we performed two permutational univariate analyses of variance (PERANOVAs), for either δ^15^N or δ^13^C and species as predictor, using the *adonis2* function implemented in the R package vegan (Oksanen et al. 2024). To identify the relationship between each metal concentration and either the feeding habitat (benthic vs. pelagic) or the trophic position, we performed a set of linear mixed effect models, with each metal concentration (log_10_ transformed) as response variable, δ^13^C or δ^15^N as fixed effect and the species as random factor, using the R package lme4 (Bates et al. 2015). Finally, we calculated the food web magnification factor (FWMF), as: FWMW = 10^b^, where b is the slope of the linear regression of each log_10_-transformed metal concentration as a function of δ^15^N. A FWMF > 1 indicates the occurrence of biomagnification, while a FWMF < 1 indicates trophic dilution (Balzani et al. 2021).

## Results

**Fig. 1:**
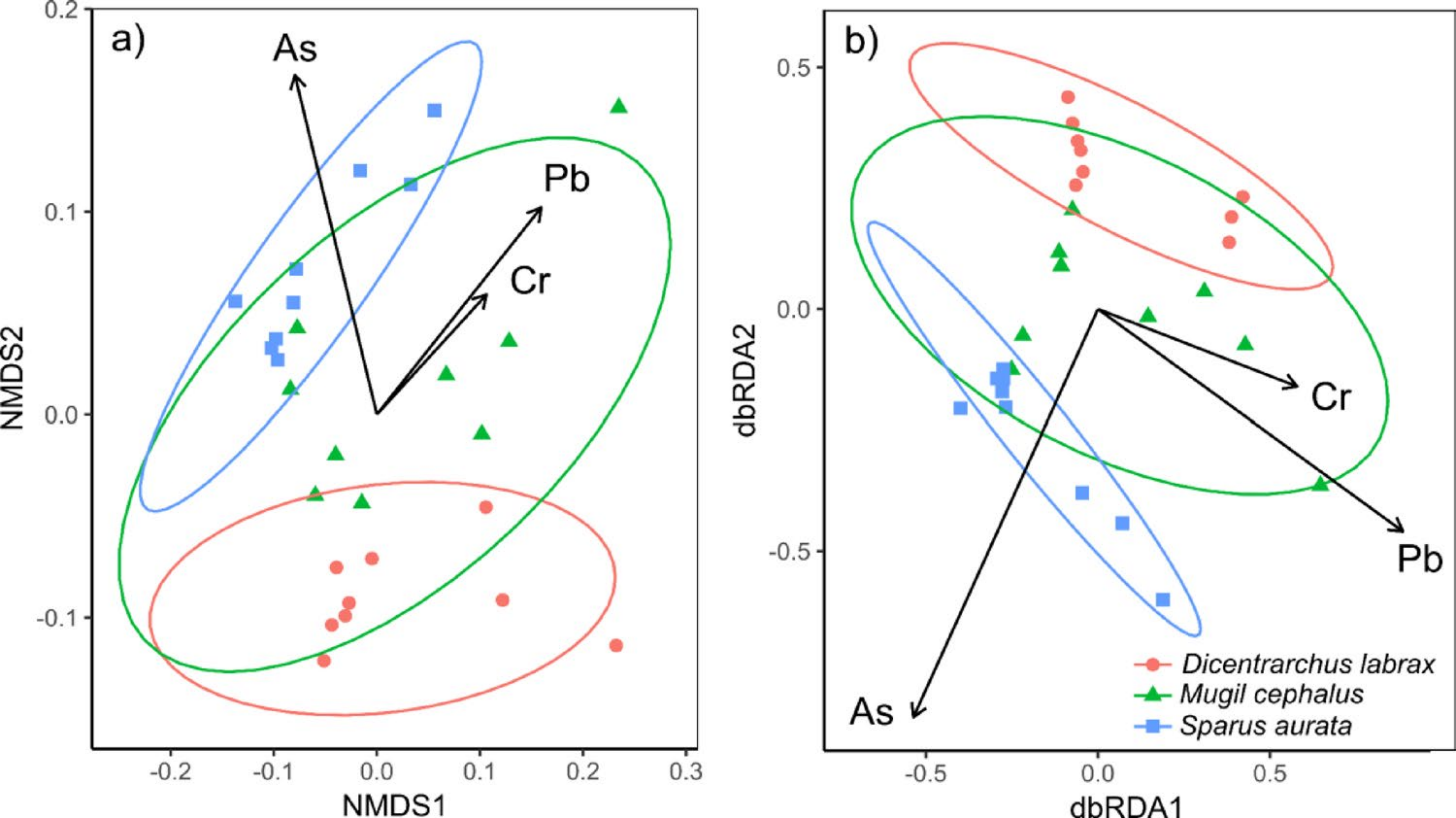
**a** Non-metric multidimensional scaling (nMDS; stress = 0.14) on the log_10_-transformed metal concentrations of the studied species. **b** db-RDA with the significant metal concentrations on the same data

The average size and metal concentration for each species is reported in Table 1. We found significant differences in the metal concentrations among species (pseudoF_2,24_ = 7.92, *P* < 0.001; Fig. 1a), with all species differing from each other (*M. cephalus* vs. *S. aurata*: *P* < 0.01; *M. cephalus* vs. *D. labrax*: *P* < 0.05; *S. aurata* vs. *D. labrax*: *P* < 0.001). The db-RDA (F_3,23_ = 44.16, *P* < 0.001; adj. R^2^ = 0.83; Fig. 1b) showed that these differences were primarily driven by differences in Pb (*P* < 0.001), As (*P* < 0.001) and Cr (*P* < 0.05) concentrations.

**Table 1:** Average (± standard error) or the total length (TL, in mm), TL (TW, in g), and metal concentrations (in µg/kg) in the sampled species

Species	TL	TW	Cr	Ni	As	Pb
*Dicentrarchus labrax*	269 (± 4.4)	187 ± (7.5)	1.54 ± (0.01)	0.56 (± 0.20)	1.06 (± 0.05)	0.25 ± (0.14)
*Mugil cephalus*	267 (± 6.4)	168 (± 13.6)	1.58 (± 0.11)	0.56 (± 0.06)	1.49 (± 0.08)	0.47 (± 0.21)
*Sparus aurata*	176 (± 1.3)	79.2 (± 1.6)	1.47 (± 0.04)	0.51 (± 0.09)	2.07 (± 0.02)	0.30 (± 0.16)

The average concentration of Cr was higher in *M. cephalus*, followed by *D. labrax* and *S. aurata* (Table 1; Figure S2). Ni average concentration was the same in *M. cephalus* and *D. labrax*, and lower in *S. aurata* (Table 1; Figure S2). As concentration was higher in *S. aurata* than *M. cephalus* and *D. labrax* (Table 1; Figure S2). Pb was higher in *M. cephalus* than *S. aurata* and *D. labrax* (Table 1; Figure S2). After log_10_-transformation, we found significant differences among species in their concentration of As only, and not for Cr, Ni, and Pb (Table 2). In particular, all species pair showed a different concentration of As (*P* < 0.001 for all).

**Table 2:** Results of Kruskal–Wallis and Mann–Whitney tests on each metal concentration (log_10_-transformed) among the three studied species (*Dicentrarchus labrax*, *Mugil cephalus*, and *Sparus aurata*)

Metal	χ^2^	*P*	*M. cephalu*s vs. *S. aurata*	*M. cephalus* vs. *D. labrax*	*S. aurata* vs. *D. labrax*
Cr	4.07	0.13	–	–	–
Ni	5.09	0.08	–	–	–
As	22.86	< 0.001	< 0.001	< 0.001	< 0.001
Pb	1.39	0.50	–	–	–

**Fig. 2:**
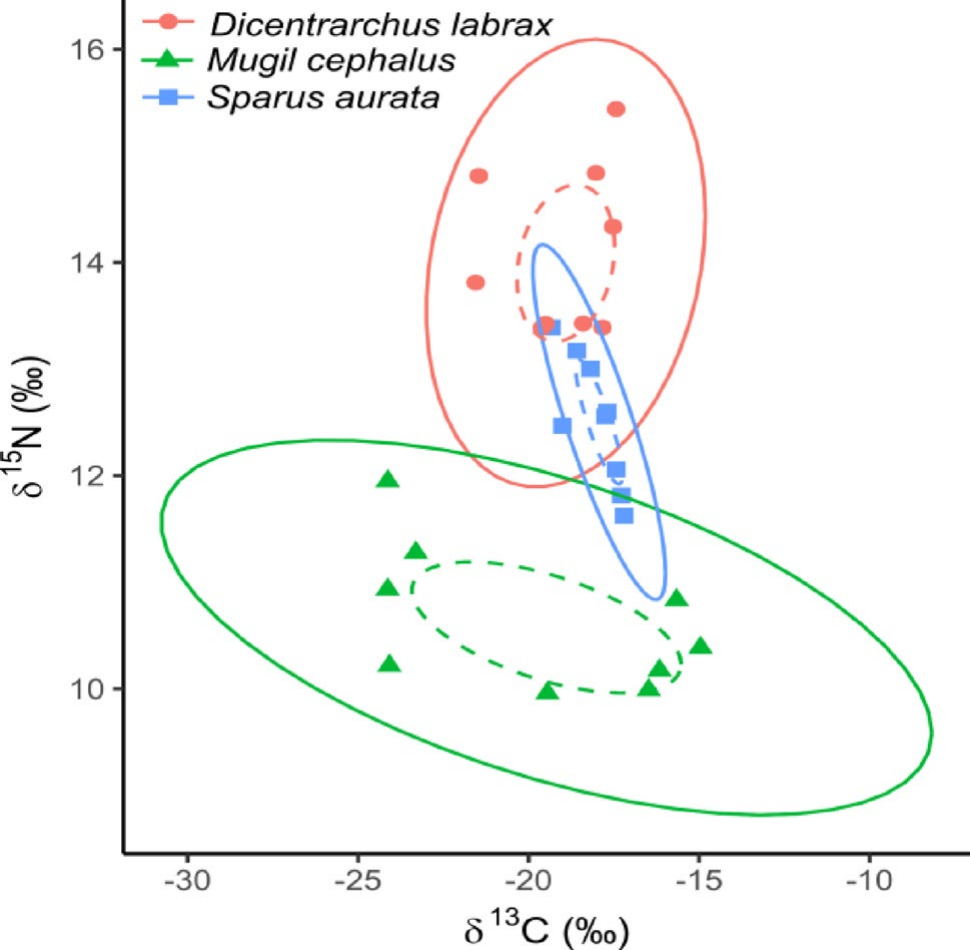
Carbon and nitrogen stable isotope standard ellipses (solid line: 95%, dashed line: 40%) of the studied species

The isotopic niches of the three species were significantly different (pseudoF_2,24_ = 4.82, *P* < 0.01; Fig. 2), with the niche of *D. labrax* being significantly partitioned from those of both *M. cephalus* (*P* < 0.05) and *S. aurata* (*P* < 0.01). On the other hand, the niches of *M. cephalus* and *S. aurata* were only marginally differentiated (*P* = 0.07). The niches were not differentiated for their δ^13^C (pseudoF_2,24_ = 1.07, *P* = 0.36), but they were for their δ^15^N (pseudoF_2,24_ = 56.70, *P* < 0.001).

Our models identified as significant only the relationship between Cr (log_10_-transformed) and δ^13^C (Table 3; Figures S3, S4), which was negative. Thus no patterns across feeding habitats or trophic position were identified. We found biomagnification for Cr and trophic dilution for the other metals, although with marginal values for Ni and Pb (Table 4).

**Table 3:** Results of the linear mixed effects models with the metal concentration (log_10_ transformed) as response variable, either carbon or nitrogen stable isotopes as predictor, and the species (*Mugil cephalus*, *Sparus aurata*, and *Dicentrarchus labrax*) as random effect

Metal	Predictor	Estimate	SE	Sum of squares	F	*P*
Cr	δ^13^C	− 0.03	0.01	0.15	4.33	< 0.05
	δ^15^N	0.01	0.03	0.01	0.20	0.73
Ni	δ^13^C	− 0.01	0.03	0.01	0.08	0.78
	δ^15^N	− 0.01	0.05	0.01	0.04	0.84
As	δ^13^C	0.02	0.01	0.07	2.79	0.11
	δ^15^N	− 0.03	0.05	0.01	0.36	0.55
Pb	δ^13^C	− 0.07	0.04	0.82	3.54	0.07
	δ^15^N	− 0.01	0.06	0.001	0.005	0.94

**Table 4:** Food web magnification factor (FWMF) for each metal in the study site

Metal	Slope	FWMW
Cr	0.004	1.01
Ni	− 0.01	0.98
As	− 0.09	0.81
Pb	− 0.004	0.99

## Discussion

Fish are widely recognized as effective bioindicators of metal contamination due to their ecological diversity, trophic connectivity, and position within aquatic food webs (Oros 2025). At the same time, they are of utmost importance for human consumption, thus assessing metal concentration and biomagnification in commercial fish species is a primary matter (Ali and Khan 2019; Madgett et al. 2021). In this study, we analyzed four metal (Cr, Ni, As, and Pb) concentrations in the muscle tissues of three commercially important fish species representing distinct feeding strategies: the omnivorous *M. cephalus*, the predatory *S. aurata*, and the top predator *D. labrax*. While the overall metal concentrations were low, significant differences were observed among species, reflecting their different ecology.

The different metal accumulation pattern among the studied species reflected their different trophic positions, but not in a linear way. Indeed, As concentrations differed among species, being highest in *S. aurata*, intermediate for *M. cephalus* and lowest in *D. labrax*. This quadratic relationship across trophic levels suggests a certain degree of trophic dilution, at least for *D. labrax*, which is a higher-trophic level, carnivorous species. On the other hand, this finding possibly reflects a greater exposure to sediment-bound forms (e.g. Zupo et al. 2019; Huang et al. 2022) due to the benthic foraging habits of *S. aurata* (Froese and Pauly 2025; Yılmaz 2005; Dural et al. 2006). As a result, we found biomagnification to occur only for Cr, with a FWMF slightly > 1 (1.01), while the other metals biodiluted across trophic levels, in line with other studies (de Souza-Araujo et al. 2022; Yang et al. 2022; but see Sun et al. 2020 for a meta-analysis). Nonetheless, despite the known coastal habits of *M. cephalus* (Chen et al. 2022), we found that the trophic niches of the three fishes were similar in their C values. As a result, we only found a negative relationship between Cr concentrations and δ^13^C, which was nonetheless mostly driven by an individual of *M. cephalus* with very high Cr concentration.

Our findings align with previous research demonstrating that metal accumulation varies among different species, depending on multiple factors, including species-specific excretion efficiency, diet, and feeding habitat (Korkmaz et al. 2019; Adams et al. 2020; Oros 2025). Similar species-specific differences in metal bioaccumulation have been reported in other freshwater and transitional systems in Türkiye (Almafrachi et al. 2025; Gümüş and Akköz 2021), where benthic-feeding fishes tended to accumulate higher metal concentrations due to direct contact with contaminated sediments and detritus-rich substrates. These regional studies support our interpretation that habitat use and foraging mode are key drivers of interspecific variation in metal uptake, and highligth the need to move beyond concentration thresholds in environmental assessments. Although tissue levels were generally below regulatory limits, species-specific patterns indicate potential for trophic transfer of contaminants, especially Cr.

Furthermore, the relatively low concentrations found in Dalyan Lake likely reflect limited point-source pollution and the predominantly rural land use around the lake. In contrast, studies from more industrialized or agriculturally intensive basins in Türkiye (e.g. Konya Plain; Almafrachi et al. 2025) report substantially higher metal concentrations in fish, driven by irrigation return flows, wastewater inputs, and sediment remobilization. Such comparisons highlight that regional differences in catchment characteristics, hydrology, and anthropogenic pressure strongly shape metal exposure pathways. In ecosystems like Dalyan Lake, where human and ecological health are tightly linked, higher trophic fish such as *D. labrax* warrant close monitoring for their role in contaminant delivery to consumers (Authman et al. 2015). Food safety regulations should be informed by ecological context, not just static metal limits. Moreover, integrating trophic position and habitat use into risk assessments allows for more accurate evaluations of exposure and bioaccumulation potential (Cresson et al. 2019; Oros 2025). This is especially relevant for commercially important species and transitional ecosystems that experience variable contamination dynamics. Our findings therefore reinforce the value of trophically informed assessments, consistent with global recommendations for improving seafood safety evaluations in impacted environments (Almafrachi et al. 2025).

Future studies in Dalyan Lake should assess metals accumulation in other species, also considering different age classes and tissues (e.g. liver, gills). Moreover, since the level of metal pollution can vary in different periods of the year (Norani et al. 2023), metal accumulation is dependent on the temperature (Ihunwo and Ibezim-Ezeani 2022), and the fish diet can vary throughout the year (Balzani et al. 2024; Berezina et al. 2024), assessing metal accumulation patterns in different seasons could be of great value, also in the light of the risks for human consumption. Finally, we should also stress that *S. aurata* in our study was smaller than the other two species, potentially representing a confounding variable. As this species changes its habitat and diet with size (Russo et al. 2007), similar-sized fishes could have shown different patterns, eventually changing the shape (linear vs. quadratic) or statistical significance, so that the patterns found in our study should be taken with caution.

## Conclusions

This study emphasizes the value of combining stable isotope analysis with contaminant analysis in evaluating pollutant transfer. Our results demonstrate that metal-specific accumulation patterns differ across fish species, with *S. aurata* having the highest As concentrations among the studied species, highlighting biomagnification of Cr and trophic dilution of Ni, As and Pb. These different patterns illustrate the complex connection between feeding, metal bioaccumulation, and species-specific physiological characteristics. Moreover, our results show that in transitional ecosystems like Dalyan Lake the feeding mode and habitat use may be more important than trophic position in determining contaminants transfer. Overall, our study provides a site-specific assessment of contaminant transfer in a socio-economically important lagoon, and highlights the need for continued, targeted monitoring to safeguard both ecosystem and human health.
